# Meltwater from West Antarctic ice sheet tipping affects AMOC resilience

**DOI:** 10.1126/sciadv.adw3852

**Published:** 2025-11-14

**Authors:** Sacha Sinet, Anna. S. von der Heydt, Henk A. Dijkstra

**Affiliations:** ^1^ Department of Physics, Institute for Marine and Atmospheric Research, Utrecht University, Utrecht, 3584 CC, Netherlands.; ^2^ Centre for Complex Systems Studies, Utrecht University, Utrecht, 3584 CC, Netherlands.

## Abstract

The Atlantic meridional overturning circulation (AMOC) and polar ice sheets are coupled tipping elements, allowing for potential cascading tipping events in which tipping is facilitated by their mutual interactions. However, while an AMOC destabilization driven by Greenland ice sheet (GIS) meltwater release is well documented, the consequences of a West Antarctic ice sheet (WAIS) tipping on the AMOC remain unclear. In the Earth system model of intermediate complexity CLIMBER-X, we perform experiments where meltwater fluxes representing plausible tipping trajectories of the GIS and WAIS are applied. We find that WAIS meltwater input can increase or decrease the AMOC resilience to GIS meltwater. Also, we find that it can completely prevent an AMOC collapse, confirming in a comprehensive model what was previously found in conceptual frameworks. Moreover, we find this stabilization to occur for ice sheet tipping trajectories that are relevant under high future greenhouse gas emission scenarios.

## Teaser

West Antarctic ice sheet tipping can prevent AMOC collapse, facilitate it, or trigger its recovery.

## INTRODUCTION

The evergrowing threat of climate change may be exacerbated by the existence of critical thresholds or tipping points within the Earth system (*1*). Once crossed, abrupt and irreversible shifts may occur in parts of the climate system known as tipping elements, of which many are crucial in maintaining the functioning and stability of the present-day climate, ecosystems, and societies.

One of these tipping elements is the Atlantic meridional overturning circulation (AMOC), a large system of ocean currents redistributing heat, salt, and nutrients between the equator and the poles. Widely sustained by the sinking of dense waters in the northern part of the Atlantic Ocean, its tipping dynamics are mainly understood in terms of the salt-advection feedback (*2*). An initial freshening of the North Atlantic results in less sinking of dense waters in this region. This leads to a weaker AMOC transporting less salt to the North Atlantic, hence amplifying the initial perturbation. Consequently, the stability of the AMOC is tightly connected to the future development of polar ice sheets, for which the mass loss (and therefore meltwater release into the ocean) is likely to increase throughout this century (*3*). In particular, the Greenland ice sheet (GIS) and West Antarctic ice sheet (WAIS) are also classified as tipping elements, and are at risk of undergoing an irreversible collapse already at the current level of warming (*4*).

On one hand, the interactions between these tipping elements set the ground for potentially dangerous domino effects referred to as cascading tipping events (*5*–*7*). In such an occurrence, the tipping of the whole system may occur while global warming only pushes one component beyond its critical threshold. A typical worst-case scenario goes as follows. In a warming world, the GIS may be destabilized via different processes such as the melt-elevation feedback (*8*, *9*), resulting in substantial meltwater release into the North Atlantic. This freshwater perturbation may then be amplified by the salt-advection feedback and result in a weakening or tipping of the AMOC (*10*). Last, the subsequent warming of the Southern Hemisphere (*11*, *12*) may trigger positive feedbacks such as the marine ice sheet instability, destabilizing the WAIS (*13*). On the other hand, this intricate system includes interactions which may result in the tipping of one component to be beneficial for another. In the aforementioned example, the AMOC weakening has been shown to imply a substantial cooling over the North Pole (*11*, *12*, *14*), which could inhibit a GIS collapse. Hence, a thorough understanding of the individual dynamics and interactions driving this coupled system of tipping elements is crucial for exploring the range of possible consequences of climate change.

A key uncertainty in how cascading tipping may or may not unfold in this system lies in how a WAIS tipping could affect the AMOC stability. The impact of Antarctic freshwater on the AMOC appears to depend on multiple processes operating at different timescales (*15*). On a decadal timescale, a decreased deep convection in the Southern Ocean can lead to an opposite, compensating response in the North Atlantic, temporarily enhancing the AMOC. However, this mechanism, known as the bipolar ocean seesaw, is not consistently reproduced in model simulations (*16*). Over a longer timescale, the northward transport of freshwater from the Southern Ocean can directly inhibit North Atlantic deep water (NADW) formation, hence weakening the AMOC. In addition, Antarctic freshwater input can influence atmospheric circulation patterns, which may both enhance or weaken the AMOC (*15*, *17*). As a result of these contrasting influences, most model studies find that the AMOC is only moderately affected by freshwater release into the Southern Ocean (*15*, *16*, *18*). Nonetheless, some studies have pointed out that such freshwater input may delay a weakening of the AMOC (*19*) or even help it to recover from a total collapse (*20*). More recently, it has been found that a collapse of the WAIS may even result in an AMOC tipping to be totally avoided (*21*, *22*). However, the AMOC response to meltwater release in the Southern Hemisphere was highly simplified, calling for investigation in models of higher complexity.

In this study, we show the different AMOC responses that can occur as a consequence of a collapse of both the GIS and WAIS using the Earth System Model of Intermediate Complexity (EMIC) CLIMBER-X version 1.0. For this, we perform experiments in which the freshwater flux is parametrized to represent ice sheet tipping trajectories occurring on a realistic timescale. In particular, we show that an AMOC tipping prevented by WAIS meltwater as found in (*21*, *22*) also exists in CLIMBER-X, and provide a mechanistic explanation of this phenomenon.

## RESULTS

Our experiments are designed to investigate the AMOC response to ice sheet tipping events occurring on a plausible timescale. However, predicting long-term (tipping) trajectories for polar ice sheets is a major challenge, which owes to uncertainties originating from ice sheet modeling, but also from future greenhouse gas emissions and the associated climate sensitivity (*3*). In general, the rate of melting increases with the level of warming, such that ice sheet tipping events occurring on the fastest timescale are typically associated with high to very high emission scenarios. It is the case of the Representative Concentration Pathway (RCP) 8.5, for which modeling studies suggest that a collapse of the GIS and WAIS could occur as fast as in 1000 (*23*) and 500 (*24*, *25*) years, respectively. These are the lower bounds proposed by recent literature, in which plausible tipping durations for the GIS were given between 1000 and 15,000 years, while tipping durations for the WAIS were given between 500 and 13,000 years (*4*). Within these relevant ranges, we design hosing experiments using the EMIC CLIMBER-X version 1.0 (see Methods), in which the meltwater forcing induced by ice sheet tipping is captured similarly to what was done in previous literature (*22*). Namely, these tipping events are parametrized only by their duration and onset delay, and inserted without compensation around the GIS and WAIS to conceptually capture a full collapse of both polar ice sheets (see Methods). These forcing trajectories are not intended to represent accurate projections of future ice sheet evolution, but rather serve as idealized scenarios to explore the range of possible AMOC responses. They are designed to conceptually capture the possibility of a full collapse of both ice sheets while systematically spanning a wide range of potential tipping durations and onset delays. This approach reflects the substantial uncertainties surrounding ice sheet tipping thresholds, timescales, and future global warming trajectories (*4*), as well as the demonstrated importance of these parameters in previous studies (*21*, *22*).

### Separate Greenland and West Antarctica meltwater impacts

**Fig. 1.**
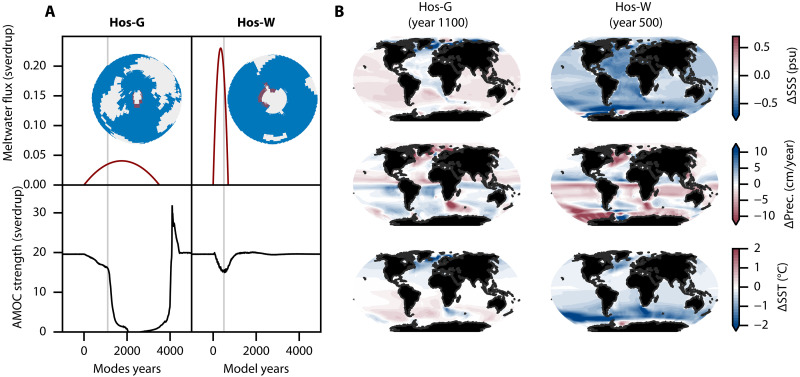
AMOC and climate response to separate GIS and WAIS meltwater fluxes. (**A**) Meltwater flux (red) and AMOC trajectory (black) in the Hos-G (left column) and Hos-W (right column) experiments, respectively. The hosing region for each experiment is displayed on the globe. (**B**) Sea surface salinity (SSS) anomaly (top), precipitation anomaly (middle), and sea surface temperature (SST) anomaly (bottom) with respect to the pre-industrial period (year 0). These are yearly averages taken at year 1100 in the Hos-G experiment (left column) and at year 500 in the Hos-W experiment (right column), indicated by gray vertical lines in (A).

Understanding the AMOC response to the combined meltwater fluxes from the GIS and WAIS requires to identify their isolated impacts. First, applying only a meltwater flux from the GIS, we find that an AMOC tipping occurs for any GIS collapse trajectory lasting between 1000 and 3900 years. In Fig. 1A (left), we show the experiment in which an AMOC tipping is induced by a GIS meltwater forcing lasting 3500 years (denoted Hos-G, see Table 1). This duration is on the order of the time it may take for the GIS to become ice-free under high to very high emission scenarios comparable to RCP8.5 or the Shared Socioeconomic Pathway (SSP) 5-8.5, with a conservative range of 1000 to about 8000 years based on model studies (*23*, *26*, *27*). From the initiation of the GIS meltwater forcing (at year 0 by convention), an AMOC weakening is found up to about year 1100, after which the AMOC rapidly collapses as the GIS meltwater forcing reaches 0.035 sverdrup (1 sverdrup = 10^6^ m^3^/s). The meltwater insertion induces a widespread freshening of the surface waters in the Northern Atlantic, as can be seen from the sea surface salinity (SSS) anomaly with respect to the pre-industrial period (year 0) just before AMOC tipping in Fig. 1B. (top left). This freshening results in a density decrease in the NADW formation regions, thereby altering deep convection, weakening the AMOC and leading to its collapse as the positive salt-advection feedback takes over. Consistent with previous literature using higher complexity models, the AMOC weakening induces both a southward shift of the intertropical convergence zone (ITCZ) (Fig. 1B, middle left), as well as a cooling and warming of the Northern and Southern Hemisphere, respectively (Fig. 1B, bottom left) (*11*, *12*, *14*). Second, from about year 3000, the AMOC slowly recovers as the GIS meltwater forcing decreases, stabilizing at pre-industrial level from about year 4500. During this phase, a very weak overturning circulation remains and, as the GIS meltwater flux decreases, initiates a positive salt-advection feedback. Namely, as salty equatorial surface waters are slowly advected northward, the progressive density increase of high-latitude North Atlantic surface waters eventually results in a reactivation of deep convection. We note that the AMOC recovery at the forcing termination is commonly found in EMICs (*28*), and that the substantial overshoot of the AMOC initial value by more than 10 sverdrup during its recovery phase was recently found in the Community Earth System Model (*29*).

**Table 1.** Summary of experiments. Each hosing experiment incorporates virtual salinity fluxes representing a full discharge of each ice sheet’s freshwater content with varying tipping durations and, when applicable, different delays between the onset of GIS and WAIS tipping (see Methods). The Hos-G and Hos-W experiments include only the representation of the GIS or WAIS tipping, respectively. The Hos-GW-Stab and Hos-GW-Reco experiments include representation of both the GIS and WAIS tipping, resulting in either AMOC stabilization (Hos-GW-Stab) or early AMOC recovery (Hos-GW-Reco).

Experiment	GIS tip. duration (years)	WAIS tip. duration (years)	Tip. delay (years)	AMOC response
Hos-G	3500	n.a.	n.a.	Collapse
Hos-W	n.a.	700	n.a.	Weakening
Hos-GW-Stab	3500	700	200	Stabilization
Hos-GW-Reco	3500	700	2600	Early recovery

Second, we isolate the effects of a meltwater flux originating from the WAIS. While no AMOC tipping is found for any WAIS meltwater forcing lasting longer than 500 years, a sustained AMOC weakening is systematically observed, following a brief initial strengthening. In Fig. 1A (right), the experiment in which an AMOC weakening of about 4.5 sverdrup is induced by a WAIS meltwater forcing lasting 700 years (denoted Hos-W, see Table 1) is shown. This forcing duration is consistent with the timescales over which a substantial to near-complete WAIS collapse is projected to occur under sustained high-emission scenarios such as RCP8.5 or SSP5-8.5, with model-based estimates ranging from just over 500 to around 1000 years (*24*, *25*). The AMOC response aligns with findings from most previous studies using models of comparable complexity (*15*, *18*, *30*). A short-term strengthening (of less than 2%) emerges about 50 years after the initiation of the WAIS meltwater forcing, which is consistent with the expected multidecadal timescale of the bipolar ocean seesaw mechanism (*15*), whereby weakened deep convection in the Southern Ocean enhances NADW production. The subsequent AMOC weakening develops on a centennial timescale, driven by the northward advection of Southern Ocean freshwater into the North Atlantic. At the weakest AMOC state (at about year 500), we find a worldwide surface water freshening especially pronounced in the North Atlantic (Fig. 1B, top right), which is well established by previous literature, and in itself suffices to explain the AMOC weakening (*15*, *16*, *18*, *30*). Moreover, the AMOC strength decrease is roughly proportional to the applied meltwater flux, although with a delay of many decades, which is about the time needed for the Southern Ocean surface freshening to propagate into the North Atlantic Ocean (*15*, *30*). Last, consistently with previous literature, we find a northward shift of the ITCZ (Fig. 1B, middle right) (*30*, *31*) as well as a pronounced cooling of the Southern Hemisphere (Fig. 1B, bottom right) (*19*, *30*, *32*).

### AMOC resilience modified by West Antarctic meltwater

**Fig. 2.**
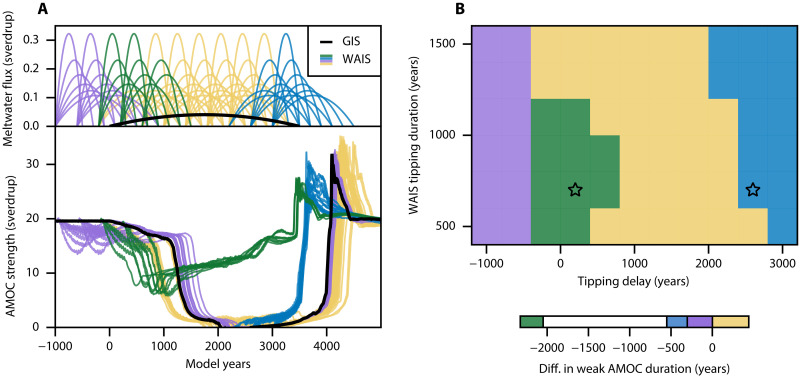
AMOC response to combined GIS and WAIS meltwater fluxes. (**A**) Trajectories of the meltwater fluxes (top) and AMOC (bottom), fixing the GIS tipping duration to 3500 years. The black trajectory is forced only by the GIS meltwater flux (Hos-G experiment, see Table 1), while these in colors include meltwater from both the GIS and WAIS. Colors represent different values of the difference in weak AMOC duration $\Delta T_{\text{weak}}$ . (**B**) Difference in weak AMOC duration $\Delta T_{\text{weak}}$ for trajectories displayed in (A), represented in the parameter plane formed by the two parameters defining the different WAIS tipping trajectories, namely, the WAIS tipping duration and the delay between the onset of the GIS and WAIS tipping events.

To understand the combined impacts of applying both GIS and WAIS meltwater fluxes on the AMOC stability, we will consider the experiment in which only the GIS meltwater forcing lasting 3500 years induced an AMOC collapse (Hos-G, see Table 1) as a baseline experiment. Then, using the same GIS meltwater forcing, we add meltwater fluxes originating from the WAIS using different values of both the WAIS collapse duration and the delay between the onset of each ice sheet tipping event. To quantify the (change in) AMOC resilience, we will use the duration of the weak AMOC state denoted $T_{\text{weak}}$ , here chosen as the time during which the AMOC strength is below 10 sverdrup [to be thought of as a characteristic return time, a classical measure of resilience in dynamical systems (*33*)]. In particular, we quantify the change of AMOC resilience induced by the WAIS meltwater flux via the following metric

$$\Delta T_{\text{weak}}=T_{\text{weak}}-T_{\text{weak}}^{0}$$ (1)

where $T_{\text{weak}}^{0}$ is the weak AMOC duration computed in the Hos-G experiment. In other words, $\Delta T_{\text{weak}}$ is the amount of years by which the weak AMOC state duration has been varied, with negative values indicating a more resilient AMOC. In Fig. 2A, the AMOC response to the GIS meltwater flux lasting 3500 years (Hos-G experiment) is shown in black, while colored lines are trajectories that also include meltwater flux from the WAIS. These colors stand for different values of $\Delta T_{\text{weak}}$ , which are also presented in the parameter space defining the different WAIS trajectories, namely, the WAIS tipping duration and delay between the onset of each ice sheet tipping event (Fig. 2B). We find that the WAIS meltwater forcing drastically affects the AMOC resilience to the GIS meltwater flux.

On one hand, a WAIS meltwater flux applied on the collapsed AMOC state, i.e., initiated roughly after year 1000, may both delay or accelerate the AMOC recovery process. In cases of an accelerated recovery (blue), the AMOC gains about 2 sverdrup as soon as the WAIS meltwater insertion starts, at a moment when the GIS meltwater flux decreases fast enough for this weak AMOC to be sustained. This early AMOC resurgence activates the positive salt advection feedback required for the AMOC recovery, which occurs up to about 500 years earlier than in the Hos-G experiment. In (*20*), a similar AMOC recovery was found in response to a freshwater forcing reaching 1 sverdrup in the Southern Ocean. The recovery was attributed to the freshening, and thus lightening, of the Antarctic intermediate water (AAIW), reestablishing a density contrast between the AAIW and NADW, which is favorable for the overturning circulation, resulting in the AMOC reactivation. Consistently, the lightening of the AAIW is also clearly found in our results (fig. S1), as can be seen in the Hos-GW-Reco experiment (blue star in Fig. 2B, see Table 1). In addition, we find a sharp decline in Antarctic bottom water (AABW) formation (indicated by a weakening of the Southern Ocean meridional overturning stream function south of 60°S) coinciding with the early AMOC resurgence in the Hos-GW-Reco experiment (fig. S2). This decline is absent in the experiment without WAIS meltwater input (Hos-G), providing a clear signature of bipolar ocean seesaw response in which decreased AABW production enhances NADW production. In the case of a delayed recovery (yellow), we find the same initial kick to the AMOC for about the duration of the WAIS meltwater forcing. However, the GIS meltwater flux is still too strong, such that the AMOC is soon attracted back to the collapsed state, delaying the recovery process. These two regimes appear to be well discriminated by the moment at which the maximum of the West Antarctic meltwater flux occurs, as we find an accelerated recovery to occur in cases where the peak WAIS meltwater flux occurs approximately after year 3000.

On the other hand, we find that a WAIS meltwater flux initiated during the active AMOC state, i.e., roughly before year 1000, systematically results in a faster AMOC weakening compared to the Hos-G experiment. This response is expected from the AMOC weakening implied by both the GIS and WAIS meltwater fluxes as found in Fig. 1A and can result in an accelerated AMOC tipping (yellow). Yet, we also find that some WAIS tipping trajectories initiated early (before or at year −600) result in a delayed AMOC collapse (purple). In a different context, similar behavior was reported in (*19*), where a substantial AMOC weakening driven by CO_2_ emissions under the RCP8.5 scenario (there defined as a drop in AMOC strength below 10 sverdrup for five consecutive years) was delayed by 35 years when projected Antarctic freshwater discharge was taken into account. Notably, we find that some WAIS tipping trajectories result in the AMOC collapse to be totally avoided (green). In these stabilization cases, the AMOC engages in a steady recovery as soon as it is decreased by a maximum 60%, resulting in an AMOC weak state duration decreased by as much as 2325 years. Qualitatively, the parameter region in which this stabilization is found appears consistent with previous conceptual research (*22*). We find that stabilization occurs for rather short and strong WAIS meltwater fluxes lasting up to 1100 years, and in cases where the peak of the WAIS meltwater flux occurs roughly 1000 years before the peak of the GIS meltwater flux. We note that the disappearance of this stabilization region can be expected from a faster GIS tipping trajectory yielding a stronger meltwater flux. To show this, we perform a similar set of experiments for a GIS meltwater flux lasting 1000 rather than 3500 years, to be thought of as a worst-case scenario (*4*, *23*). In this case, while the earlier recovery and tipping regimes are still found, WAIS meltwater fluxes are only able to marginally delay the AMOC tipping, while the stabilization regime totally disappears within the tested parameter range (fig. S3). Last, considering the results using a GIS tipping duration of 3500 years (Fig. 2), the AMOC stabilization occurs for ice sheet tipping trajectories that are relevant considering high to very high future emission pathways. An upper bound for the tipping point of both the GIS and WAIS at 3°C above pre-industrial levels was proposed by recent literature (*4*). This suggests that the tipping of both ice sheets is likely to occur within the next century in the case of SSP3-7.0 or higher emission scenarios, under which the ice sheet tipping timescales used in our experiments are most plausible. This would translate into a delay between the initiation of both tipping events of maximum a century which, in our experiments, is the most favorable situation for the stabilization to occur, requiring a rather fast WAIS tipping lasting no more than 1100 years.

### Mechanisms of AMOC stabilization

To understand how the stabilization process occurs, we proceed to a comparison between two experiments. On one hand, we consider the Hos-G experiment (see Table 1) in which only a GIS meltwater flux lasting 3500 years yields an AMOC collapse. On the other hand, we consider the Hos-GW-Stab experiment (green star in Fig. 2B, see Table 1) in which the same GIS meltwater flux is applied, while a WAIS meltwater flux lasting 700 years and initiated 200 years after the GIS meltwater flux yields an AMOC stabilization.

To explain the AMOC stabilization, a first possible mechanism is the bipolar ocean seesaw (*15*), wherein a reduction in AABW formation caused by freshwater input into the Southern Ocean leads to a strengthening of the AMOC, driven by a compensatory enhancement of NADW production. We find that freshwater input from the WAIS causes a near halving of AABW production in the Hos-GW-Stab experiment (fig. S4), establishing a background state that could support a stronger AMOC. Also, as found earlier when considering the WAIS meltwater flux in isolation (Hos-W experiment, see Table 1), we find a short-lived signature of the bipolar ocean seesaw within the first few decades, during which a marginal AMOC strengthening is observed (*15*, *18*, *30*). However, during the stabilization, the transient AMOC response in the Hos-GW-Stab experiment is inconsistent with the bipolar ocean seesaw mechanism. Specifically, variations in AABW formation and AMOC strength remain mostly positively correlated until around the year 2500 (fig. S4 and movie S1), which is the opposite of what the bipolar ocean seesaw would imply. In line with previous studies (*15*, *18*, *30*), this suggests that on the multicentennial timescale on which the stabilization occurs, the dominant influence of WAIS freshwater on the AMOC is not the bipolar ocean seesaw mechanism, but instead the AMOC weakening due to the northward advection of freshwater from the Southern Ocean into the North Atlantic.

**Fig. 3.**
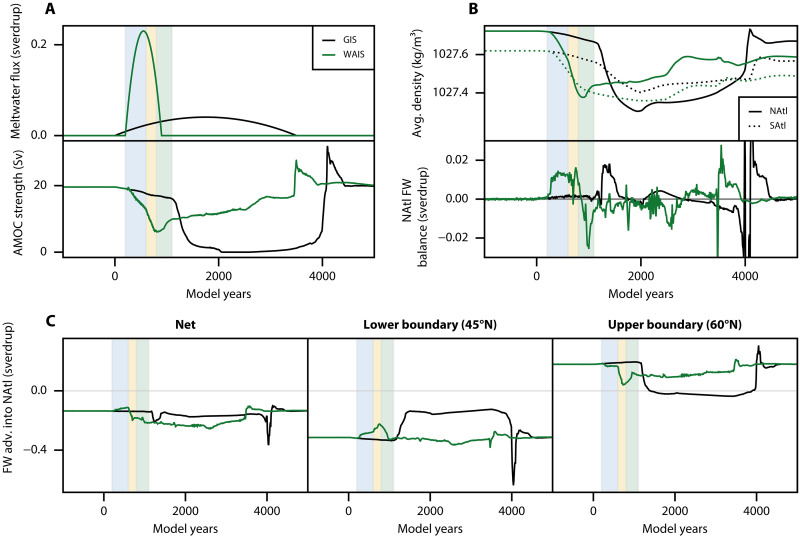
AMOC stabilization driven by West Antarctic meltwater. (**A**) Trajectory of the meltwater fluxes and AMOC strength in the Hos-G (black) and Hos-GW-Stab (green) experiments, the latter including both the GIS and WAIS meltwater fluxes. (**B**) Average density of the NAtl and SAtl boxes (top) and freshwater balance of the NAtl box (bottom), displayed for both the Hos-G (black) and Hos-GW-Stab (green) experiments. (**C**) Net freshwater advection into the NAtl box (left), along with the advection into the NAtl box at its lower (middle) and upper (right) boundaries. These are displayed for both Hos-G (black) and Hos-GW-Stab (green) experiments. In all panels, light blue, yellow, and green vertical bands indicate the first, second, and third stages of the AMOC stabilization, respectively.

Therefore, we propose a mechanism for AMOC stabilization that reconciles both its observed weakening and eventual stabilization. For the purpose of our analysis, we compute the average density in two Atlantic Ocean regions: a northern box (NAtl, 45°–60°N) and a southern box (SAtl, 35°–20°S). The density in the NAtl box reflects the potential for deep convection in the North Atlantic, while the density difference between the NAtl and SAtl boxes quantifies the meridional density gradient across the Atlantic basin, an observable correlated with the AMOC strength. In addition, we compute the full freshwater balance of the NAtl box (Fig. 3 and fig. S5, see Methods). In this context, the stabilization process can be understood in the following three stages.

**Fig. 4.**
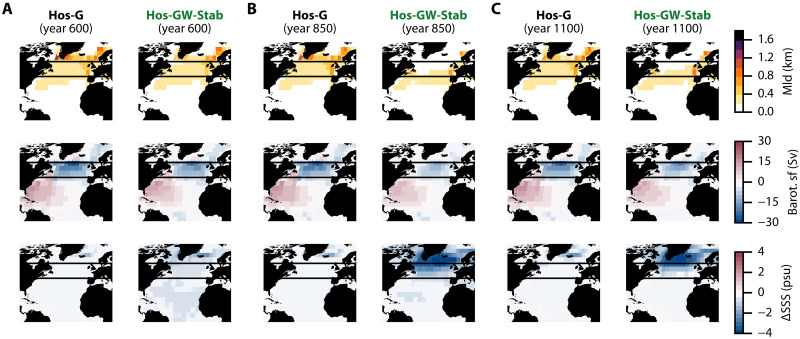
AMOC diagnostics at the end of each stabilization stage. (**A**) Diagnostics of the maximum monthly mean mixed layer depth (Mld), barotropic stream-function, and sea surface salinity (SSS) anomaly with respect to the pre-industrial period (year 0) at the end of the first stabilization stage (year 600) for the Hos-G (left) and Hos-GW-Stab (right) experiments. The upper and lower bounds of the NAtl box are represented as horizontal black lines. (**B**) Same as (A) but at the end of the second stabilization stage (year 800). (**C**) Same as (A) but at the end of the third stabilization stage (year 1100).

In the first stage (between years 200 and 600, light blue band in Fig. 3), we find an accelerated weakening of the AMOC in the Hos-GW-Stab experiment (Fig. 3A, bottom). As previously explained, this mostly originates from the meltwater discharge around the WAIS being advected into the Atlantic Ocean, and in particular into the North Atlantic. This northern freshwater transport is evident from the increase of advective freshwater import at the lower boundary of the NAtl box (Fig. 3C, middle), which can be further identified as being advected northward by the overturning circulation itself (fig. S5, bottom middle). At the end of the first stage (Fig. 4A), the increased surface freshening in the North Atlantic compared to the Hos-G experiment is clear, along with a weakening of both deep convection and the gyre circulation, as can be seen from the maximum monthly mean mixed layer depth and barotropic stream function, respectively. We note that the weakening of the gyre circulation as the AMOC strength decreases is also found in the higher hierarchy of models (*34*).

In the second stage (between years 600 and 800, light yellow band in Fig. 3), the Hos-GW-Stab experiment yields a sharp decline of the AMOC strength as convection in high latitudes collapses. This occurs as the gyre circulation, which normally transports the freshwater from the North Atlantic to lower latitudes, is now too weak for advecting the increasing freshwater buildup forming around the GIS southward (Fig. 3C, right, and fig. S5, top and bottom right). This leads to accumulation of freshwater around Greenland, thereby decreasing the surface water density there, and stopping deep convection as well as weakening the gyre circulation further. At the end of the second stage, the collapse of deep convection in the subpolar region, weakening of the gyres as well as freshwater buildup around the GIS, are established (Fig. 4B).

In the third stage (between years 800 and 1100, light green band in Fig. 3), the AMOC begins to recover and stabilizes to a weak but active state. On one hand, we find that this recovery from year 800 correlates with a sharp density increase in the North Atlantic (Fig. 3B, top). This occurs as, at this point, the weak gyre circulation only advects a fraction of the GIS freshwater southward (Fig. 3C, right, and fig. S5C, bottom right), resulting in most of this freshwater to accumulate at high latitudes, thereby preserving the deep convection that continues to occur south of 55°N (Fig. 4C). Meanwhile, as the meltwater flux originating from the WAIS weakens, the northern advection of freshwater from the Southern Ocean diminishes (Fig. 3C, middle). As seen from the freshwater balance (Fig. 3B, bottom), this results in a net freshwater export out of the NAtl box, meaning an increasing salinity and hence density there (Fig. 3B, top), which favors the recovery of deep convection in the North Atlantic. On the other hand, as the fraction of the increasing GIS freshwater flux reaching the NAtl box is advected southward, the density of the SAtl box decreases (Fig. 3B, top), leading to an increasing meridional density gradient within the Atlantic Ocean. In summary, both the increase of density in the North Atlantic and the increasing meridional density gradient between the North and South Atlantic contribute to maintaining the AMOC in a weaker, but active state. Notably, in the same model, this weak AMOC state characterized by convection occurring south of 55°N (Fig. 4C) has already been found in (*35*), where it was shown to be stable at these higher values of the northern meltwater flux.

## DISCUSSION

Our results shed light on the impact that a future GIS and WAIS tipping may have on the AMOC. While the destabilizing influence of a GIS meltwater discharge is found consistently with previous literature, we identify qualitatively different regimes that can occur when a WAIS tipping is also represented. In the context of a GIS-induced AMOC tipping, our experiments demonstrated that WAIS meltwater can have a variety of influences on the AMOC in CLIMBER-X, some of which have previously been described in the literature. For example, we reproduced previous findings in which WAIS meltwater applied on a collapsed AMOC can trigger its recovery (*20*). We found regimes in which the AMOC weakening could be delayed (*19*), which in our case requires a WAIS meltwater flux initiated centuries before the GIS meltwater input. We also found that WAIS meltwater can decrease the AMOC resilience to GIS freshwater by both accelerating the AMOC tipping or delaying its recovery. Last, we demonstrate that WAIS meltwater is able to totally prevent the AMOC collapse, an outcome initially found in conceptual studies (*21*, *22*) and now in a comprehensive climate model.

We also provide a mechanistic understanding of this stabilization phenomenon. In the first stage, the northward meltwater export from the WAIS results in a faster AMOC weakening leading, in the second stage wherein the GIS meltwater flux becomes stronger, to an early collapse of the circulation at high latitudes. This sets the AMOC in a weaker but more resilient stable state, in which the GIS meltwater accumulating around the GIS has only a limited impact on convection regions active at lower latitudes. This alternative equilibrium that was already found earlier (*35*) leads, in the third stage, to the AMOC stabilization and eventual recovery as the GIS forcing decreases. Similarly to previous studies (*21*, *22*), stabilization occurs for a rather fast collapse of the WAIS lasting up to 1100 years, and in cases where the peak of WAIS meltwater flux occurs about a thousand years before the peak of GIS meltwater flux.

The wide range of WAIS tipping trajectories explored in this study can be interpreted in light of both future and past climate. In terms of future emission scenarios and in the case of a tipping of both polar ice sheets, plausible tipping points proposed by literature (*4*) would result in both ice sheets to collapse within the 21st century for high emission scenarios SSP3-7.0 to SSP5-8.5, or within the next 300 years for milder emission scenarios such as SSP2-4.5 when considering extended SSP scenarios (*36*). Hence, any of these scenarios would result in a delay between the initiation of ice sheet tipping at both poles of at most a few centuries, resulting in most likely future scenarios to be either an accelerated or prevented AMOC collapse. Yet, the rather fast GIS tipping lasting 3500 years used in our experiments makes it safer to interpret our results in the context of high to very high emission scenarios. In this case, a fast and early collapse of the WAIS on the (sub)millennial timescale is possible, resulting in the AMOC stabilization phenomenon being a credible possibility. Meanwhile, scenarios of a WAIS collapse acting on a tipped AMOC (i.e., for tipping delays greater than 2000 years) are relevant in the context of paleo-climate. We have found that the AMOC recovery can be triggered by the WAIS meltwater discharge, which was already found in a model of similar complexity and given as potential explanation of the Meltwater pulse 1A event leading to the termination of the last glacial period (*20*). Last, another study showed that the stable AMOC weak state found in the stabilization process corresponds to stadial-like conditions during Dansgaard-Oeschger events (*37*). However, whereas this weak state was found via a variation of ice sheet configuration, greenhouse gas concentration, and northern freshwater flux, our results show that it can be obtained and explained solely via combined ice sheet tipping events.

There are limitations inherent to the simplifications of our study, which require the quantitative results to be interpreted with care. The simplified representation of GIS and WAIS meltwater fluxes trajectories, as well as their distribution in space, were not designed to accurately represent future ice sheet tipping trajectories, and hence do not capture many real-world processes. This includes, for example, basal melting beneath ice shelves and the associated ocean heat loss through ice-ocean heat exchange (*38*), or the meltwater released by drifting icebergs (*39*), which modify the spatial and temporal characteristics of freshwater input to the ocean. Yet, these trajectories capture events taking place in the region of interest, and allow to simply and systematically represent the plausible meltwater forcing rates and magnitudes that, according to literature (*4*), may be involved in future ice sheet tipping events. Also, our experiments did not include interactions from the ocean to the ice sheets, such that impacts of an AMOC collapse on the GIS and WAIS are not present. The subsequent Northern Hemisphere cooling and Southern Hemisphere warming (*11*, *12*) would likely result in an inhibition and acceleration of the GIS and WAIS meltwater fluxes, respectively (*7*). Whereas it is clear that a less brutal GIS meltwater flux may render an AMOC tipping less likely, a faster and/or earlier WAIS tipping may, as we have seen, affect the AMOC resilience. Last, the critical value of GIS freshwater flux leading to an AMOC collapse (0.035 sverdrup in the Hos-G experiment) is relatively low compared to other studies, for which lower bounds are rather given at about 0.1 sverdrup. For instance, a study using a more complex model in a comparable experimental setup suggested a critical range of 0.06 to 0.16 sverdrup (*32*). Therefore, our experiments should not be directly interpreted as evidence that GIS tipping alone would be sufficient to induce an AMOC collapse.

Our results clearly demonstrate that the AMOC stabilization driven by WAIS meltwater fluxes is not only present in conceptual models, but can also be found in EMICs. Although the exploratory nature of our study did not permit the use of high-resolution models such as those in the sixth phase of the Coupled Model Intercomparison Project (CMIP6), the use of the well-established EMIC CLIMBER-X provides confidence in the representation of the simulated processes. This is particularly true given the fact that the climate response to meltwater fluxes in both hemispheres aligns well with other model studies, also involving climate models of higher complexity. This is encouraging for further studies using models of both similar and higher complexity, which are still needed to quantify uncertainties and provide insights into the robustness of the mechanisms involved in the stabilization process.

The crucial impact of melting rates on AMOC resilience highlighted in our study underscores the necessity of producing long-term projections of ice sheet evolution under diverse emission scenarios, as well as developing advanced coupled models that integrate ice sheet dynamics, oceanic processes, and their interactions. In addition, reliable observations are crucial not only to monitor the rate of change in meltwater fluxes but also to diagnose the AMOC state and, given recent advances in early warning signals (*14*), proximity to tipping.

Our results show that diverse influences of WAIS meltwater on the AMOC can coexist in a single model. While we emphasize on the beneficial role that a WAIS tipping can have, such a marked event is far too dangerous to bet on given its many severe consequences including, for example, a total contribution to global sea level rise of up to 4.3 m (*40*). Hence, it does not undermine the need for mitigation efforts necessary to avoid any tipping event in the first place. Nonetheless, the profound implications of a prevented AMOC tipping driven by WAIS meltwater for future climate and necessary adaptation make it essential to consider, investigate further, and motivate efforts to include a representation of ice sheet meltwater input into the ocean in CMIP class models.

## METHODS

### The climate model

We use the EMIC CLIMBER-X version 1.0, which is extensively described in (*41*). It includes the semi-empirical statistical-dynamical atmosphere model SESAM, the 3D frictional-geostrophic balance ocean model GOLDSTEIN, the sea ice model SISIM, and the land model PALADYN, which are all discretized on a 5° × 5° horizontal grid and are systematically ran on a yearly time resolution. Each simulation branches off from the end of a pre-industrial control run at which the AMOC is in a monostable regime.

CLIMBER-X performs in many aspects similarly to state-of-the-art CMIP6 models under a variety of forcings and boundary conditions (*41*) and offers a suitable and efficient framework for investigating the long-term stability of the AMOC. The simulated present-day AMOC overturning profile at 26°N aligns well with observations, falling within the CMIP6 model range, with North Atlantic deep convection patterns that are consistent with ocean reanalysis. The AMOC stability in CLIMBER-X has been extensively studied (*35*), showing the typical AMOC hysteresis behavior that is also found across the hierarchy of models (*28*, *29*). Moreover, CLIMBER-X has recently been shown to capture Dansgaard-Oeschger–like variability under midglacial conditions (*37*), underscoring its ability to capture key processes governing long-term AMOC dynamics and stability.

### The meltwater forcing

An idealized representation of collapsing ice sheets is introduced, analogous to what was done in (*22*). Namely, we use meltwater flux trajectories that are parabolic in time, allowing to conceptually capture the total duration of each ice sheet tipping event, as well as their delay in time. These trajectories are noted $F_{\text{GIS},\text{WAIS}}\left(t\right)$ for the GIS and WAIS meltwater flux, respectively, and are given by

$$F_{\text{GIS}}\left(t;P_{\text{GIS}}\right)=-\frac{6V_{\text{GIS}}}{P_{\text{GIS}}^{3}}t\left(t-P_{\text{GIS}}\right)$$ (2)

if $0<t<P_{\text{GIS}}$ and zero otherwise, and

$$F_{\text{WAIS}}\left(t;P_{\text{WAIS}},\Delta t\right)=-\frac{6V_{\text{WAIS}}}{P_{\text{WAIS}}^{3}}\left(t-\Delta t\right)\left(t-\Delta t-P_{\text{WAIS}}\right)$$ (3)

if $0<t-\Delta t<P_{\text{WAIS}}$ and zero otherwise. There, *t* is the time in years, Δt is the delay between the onset of the GIS and WAIS tipping, $P_{\text{GIS},\text{WAIS}}$ is the duration of each ice sheet tipping event, while $V_{\text{GIS},\text{WAIS}}$ is the freshwater content of the GIS and WAIS, as given in (*21*). By convention, the GIS collapse is always initiated at year *t* = 0. This meltwater forcing is applied as a surface, virtual salinity flux without compensation, capturing a full collapse of both the GIS and WAIS. Meltwater fluxes are uniformly spread among grid cells surrounding the GIS and WAIS, as shown in Fig. 1A. These regions have been chosen as follows. In both cases, meltwater flux is applied to the first ocean cells directly adjacent to ocean-free cells covering the GIS or WAIS ice sheet regions. In the case of GIS hosing region (Fig. 1A, left), such cells are only selected south of 80.0°N, informed by the procedure depicted by (*42*) and used in the framework of the North Atlantic Hosing Model Intercomaprison Project (NAHosMIP) (*10*). In the case of the WAIS hosing region (Fig. 1A, right), such cells are selected between 167.5°E and 27.5°W, i.e., approximately from the beginning of the Ross ice shelf to the end of the Ronne-Filchner ice shelf.

### The freshwater balance

The freshwater budget over the NAtl box is defined as the time derivative of the total amount of freshwater it contains, here denoted *W*, and given by

$$\frac{dW}{dt}=\Delta F_{\text{adv}}+\Delta F_{\text{diff}}+F_{\text{surf}}$$ (4)

Here, $F_{\text{surf}}$ is the total surface freshwater flux into the box including, for example, precipitation and runoff. Meanwhile, $F_{\text{adv},\text{diff}}$ refer to the total northern freshwater transports within the Atlantic due to advection and diffusion at some given latitude, such that $\begin{array}{c} \Delta F_{\text{adv},\text{diff}}=F_{\text{adv},\text{diff}}\;\left(45^{\circ }N\right)-F_{\text{adv},\text{diff}}\;\left(60^{\circ }N\right) \end{array}$ describes the variation of freshwater content within the NAtl box due to each component. Last, the overturning and azonal components result from a commonly used decomposition of the advective transport [see, e.g., (*43*)], providing the contribution of both the overturning and gyre circulation to the advective transport.
