# Supplementary material for: Meltwater from West Antarctic ice sheet tipping affects AMOC resilience
Source: Sci Adv. 2025 Nov 14;11(46):eadw3852. doi: 10.1126/sciadv.adw3852 (PMC12617517; doi:10.1126/sciadv.adw3852)
Supplement: Supplementary file 1 — Figs. S1 to S5 Legend for movie S1 [file sciadv.adw3852_sm.pdf]

Science Advances
AAAS

Supplementary Materials for

Meltwater from West Antarctic ice sheet tipping affects AMOC resilience

Sacha Sinet *et al.*

Corresponding author: Sacha Sinet, s.a.m.sinet@uu.nl

Sci. Adv. **11**, eadw3852 (2025)
DOI: 10.1126/sciadv.adw3852

The PDF file includes:

Figs. S1 to S5
Legend for movie S1

Other Supplementary Material for this manuscript includes the following:

Movie S1

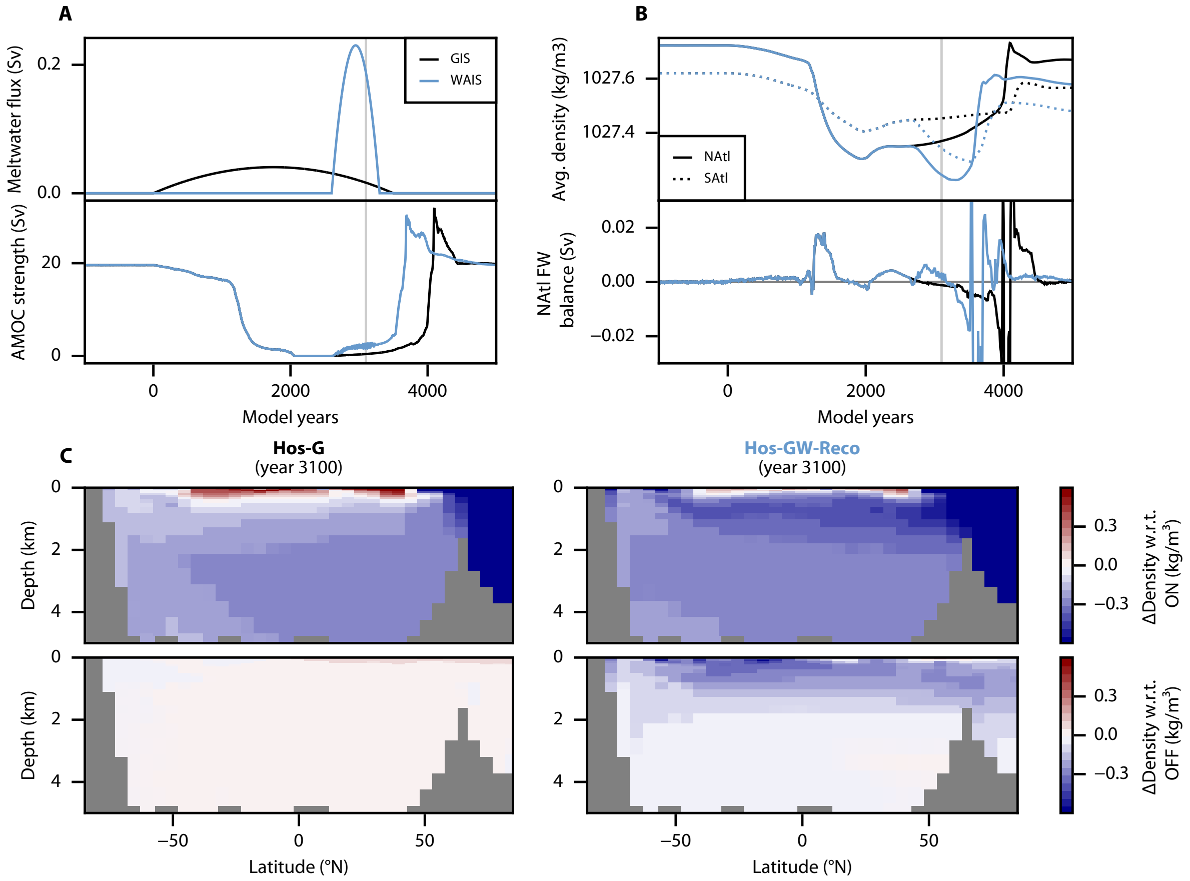

Figure S1: **AMOC recovery induced by West Antarctic meltwater – AAIW lightening.** (**A**) Trajectory of the meltwater fluxes and AMOC strength in the Hos-G (black) and Hos-GW-Reco (blue) experiments, the latter including both the GIS and WAIS meltwater fluxes. (**B**) Average density of the NAtl and SAtl boxes (top) and freshwater balance of the NAtl box (bottom), displayed for both the Hos-G (black) and Hos-GW-Reco (blue) experiments. (**C**) Yearly averaged, zonally integrated density anomaly in the Atlantic Ocean and the Atlantic sector of the Southern Ocean (taken between 70°W and 20°E), with respect to the AMOC ON (active) and OFF (collapsed) states (top and bottom, respectively) for both the Hos-G and Hos-GW-Reco experiments (left and right, respectively). These are displayed at year 3100, depicted by the vertical grey line in panels A and B.

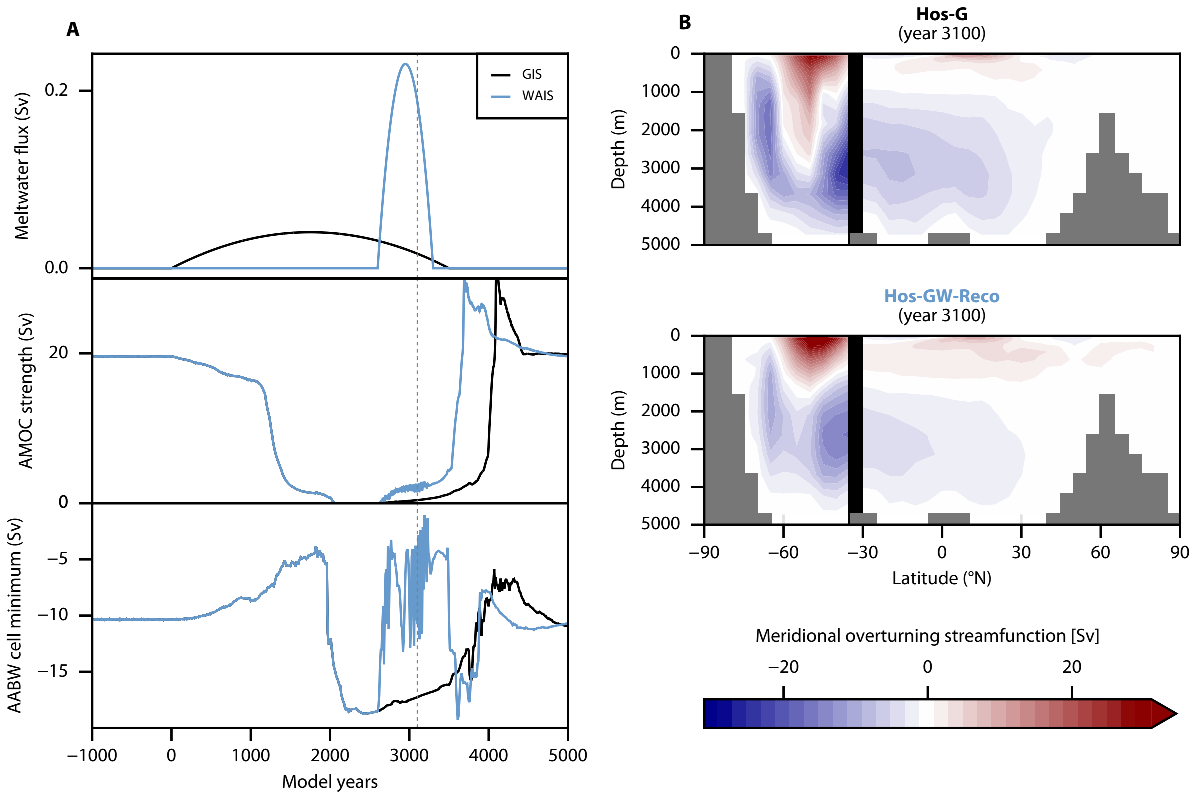

Figure S2: **AMOC recovery induced by West Antarctic meltwater – bipolar ocean seesaw.** (**A**) Trajectory of the meltwater fluxes, AMOC strength and AABW cell minimum (i.e. minimum value of the Southern Ocean meridional overturning streamfunction south of 60°S). The AMOC strength and AABW cell minimum are used as indicators of NADW and AABW production, respectively. These are represented in the Hos-G (black) and Hos-GW-Reco (blue) experiments, the latter including both the GIS and WAIS meltwater fluxes. (**B**) Yearly averaged value of the meridional overturning streamfunction in both the Southern Ocean and Atlantic Ocean, separated by a black vertical column. These are computed at year 3100 (vertical dashed line in A) in the Hos-G (top) and Hos-GW-Reco (bottom) experiments.

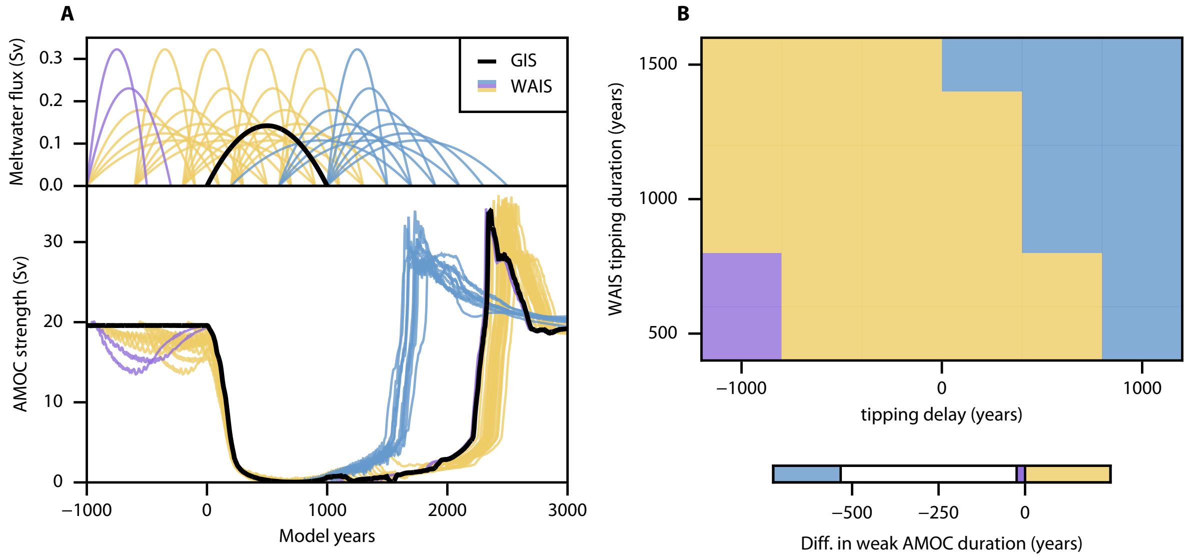

Figure S3: **AMOC response to combined GIS and WAIS meltwater fluxes for a shorter GIS tipping trajectory.** (**A**) Trajectories of the meltwater fluxes (top) and AMOC (bottom), fixing the GIS tipping duration to 1000 years. The black trajectory is forced only by the GIS meltwater flux, while these in colours include meltwater from both the GIS and WAIS. Colours represent different values of the difference in weak AMOC duration ΔT_{weak}. (**B**) Difference in weak AMOC duration ΔT_{weak} for trajectories displayed in A, represented in the parameter plane formed by the two parameters defining the different WAIS tipping trajectories, namely the WAIS tipping duration and the delay between the onset of the GIS and WAIS tipping events

.

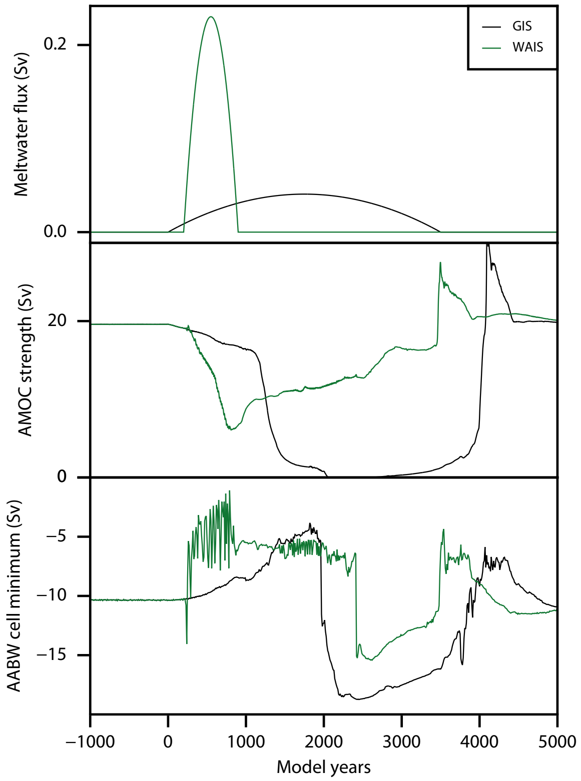

Figure S4: **AMOC stabilization and AABW production..** Trajectory of the meltwater fluxes, AMOC strength and AABW cell minimum (minimum value of the Southern Ocean meridional overturning streamfunction south of 60°S). The AMOC strength and AABW cell minimum are used as indicators of NADW and AABW production, respectively. These are represented in the Hos-G (black) and Hos-GW-Stab (green) experiments, the latter including both the GIS and WAIS meltwater fluxes.

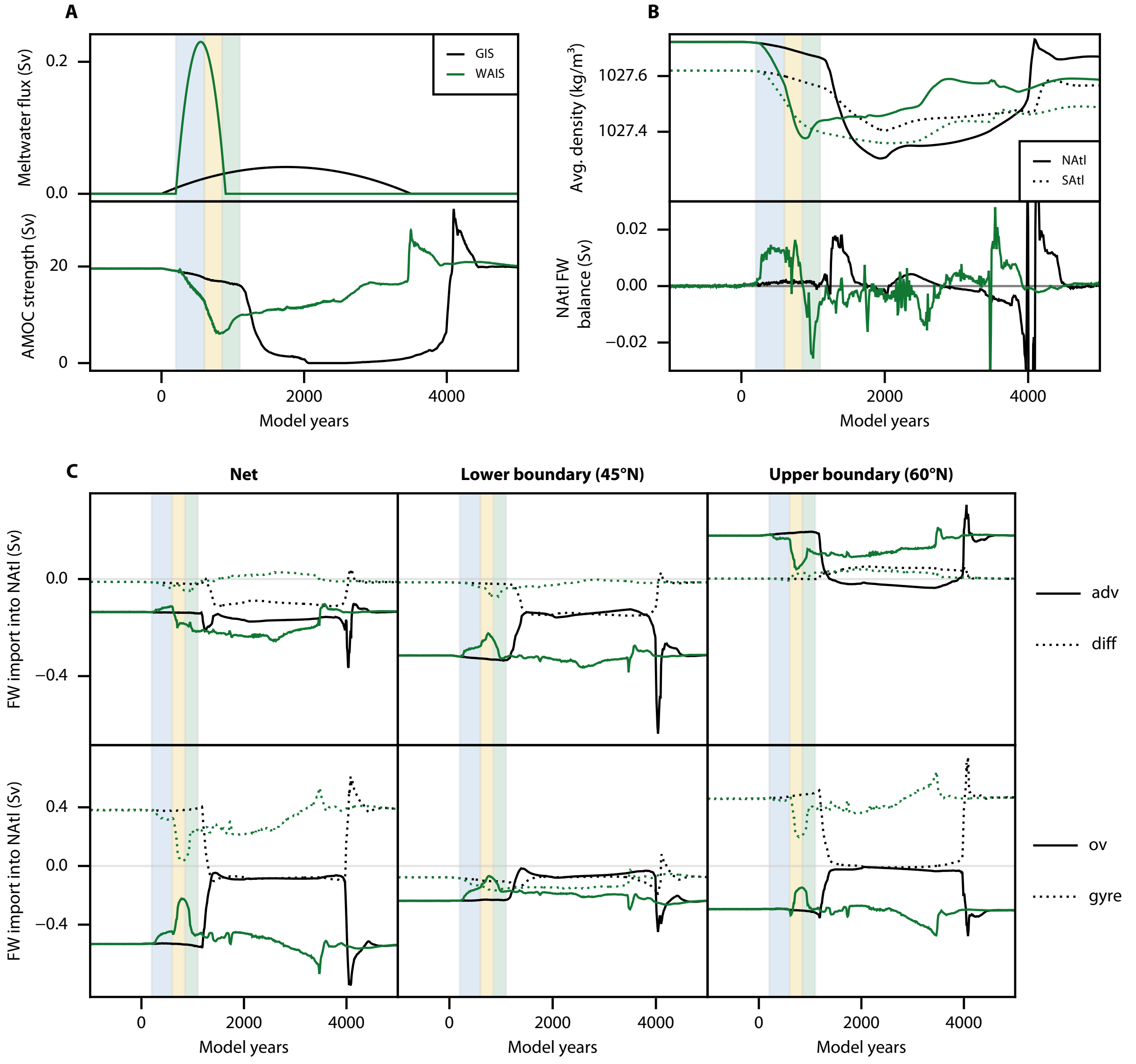

Figure S5: **AMOC stabilization driven by West Antarctic meltwater with more detailed freshwater contributions.** (**A**) Trajectory of the meltwater fluxes and AMOC strength in the Hos-G (black) and Hos-GW-Stab (green) experiments, the latter including both the GIS and WAIS meltwater fluxes. (**B**) Average density of the NAtl and SAtl boxes (top) and freshwater balance of the NAtl box (bottom), displayed for both the Hos-G (black) and Hos-GW-Stab (green) experiments. (**C**) Net freshwater import into the NAtl box (left), along with the import into the NAtl box at its lower (middle) and upper (right) boundaries. It includes the contributions of the advection and diffusion (top) and contributions from both the overturning and gyres (bottom). These are displayed for both Hos-G (black) and Hos-GW-Stab (green) experiments. In all panels, light blue, yellow and green vertical bands indicate the first, second and third stages of the AMOC stabilization, respectively.

Movie S1. AMOC stabilization driven by West Antarctic meltwater — evolution of Southern Ocean and Atlantic overturning.

(A) Trajectories of meltwater fluxes, AMOC strength, and AABW cell minimum in the Hos-G (black) and Hos-GW-Stab (green) experiments, the latter including meltwater fluxes from both the GIS and WAIS. The AABW cell minimum is quantified as the minimum of the Southern Ocean meridional overturning cell south of 60°S. (B) Southern Ocean and Atlantic meridional overturning streamfunctions at the model year indicated by the dashed grey vertical line in A.
